# Towards quantifying plasmid similarity

**DOI:** 10.1099/mgen.0.001290

**Published:** 2024-09-12

**Authors:** William Matlock, Liam P. Shaw, Samuel K. Sheppard, Edward Feil

**Affiliations:** 1 Nuffield Department of Medicine, University of Oxford, Oxford, UK; 2 Department of Biology, University of Oxford, Oxford, UK; 3 Milner Centre for Evolution, Department of Life Sciences, University of Bath, Bath, UK

**Keywords:** antimicrobial resistance, genomic epidemiology, mobile genetic elements

## Abstract

Plasmids are extrachromosomal replicons which can quickly spread resistance and virulence genes between clinical pathogens. From the tens of thousands of currently available plasmid sequences we know that overall plasmid diversity is structured, with related plasmids sharing a largely conserved ‘backbone’ of genes while being able to carry very different genetic cargo. Moreover, plasmid genomes can be structurally plastic and undergo frequent rearrangements. So, how can we quantify plasmid similarity? Answering this question requires practical efforts to sample natural variation as well as theoretical considerations of what defines a group of related plasmids. Here we consider the challenges of analysing and rationalising the current plasmid data deluge to define appropriate similarity thresholds.

## Introduction

Plasmids are extrachromosomal replicons. Many plasmids are conjugative, encoding a pilus which allows transfer into a new host cell [1]. In addition to their replication, maintenance, and transfer machinery, plasmids carry a diverse array of accessory genes, including those encoding antimicrobial resistance (AMR), virulence factors (VFs), and metabolic capabilities, thereby influencing the phenotype of their bacterial hosts [2]. In clinical contexts, their ability to spread AMR genes between species can lead to outbreaks of resistant pathogens. Plasmids can be grouped based on their replicons (replicon typing) [3], relaxases (MOB typing) [4], or overall genetic similarity (for example, with plasmid taxonomic units [PTUs] [5] or shared *k*-mer content [6]). However, these classification schemes are typically too broad (and often conflicting [7] when the aim is to infer recent transfer events).

Genomic epidemiology was developed for chromosomes, where high genetic similarity is indicative of recent shared ancestry. Established methods use variations in the core genome (e.g. alleles, shared polymorphisms, or fixed differences) [8]. Other methods also consider accessory genome variation [9,10]. However, plasmids are different to chromosomes: they are smaller, transferable, and flexible. Different methodology to infer recent transfer might be required. Here, we will review the main mechanisms by which plasmids evolve, and how these pose challenges for epidemiology.

## Mutations as a measure of relatedness

The concept of a plasmid ‘backbone’ was first introduced by Smith and Thomas in 1987 to describe stretches of homologous sequence shared between diverse IncP plasmids [11]. The backbone had conserved synteny but was occasionally interrupted by non-homologous stretches, often unique to individual plasmids. More recent work shows that most closely-related plasmids contain a common backbone [12]. The backbone contains ‘essential’ genes including those for plasmid maintenance and conjugative transfer, but non-essential core genes may also be included due to their high frequency [13]. The remaining genes carried by plasmids are ‘cargo’ (or ‘accessory’) genes. These are involved in the other functions that plasmids can encode, e.g. genes for AMR, niche adaptation, or smaller mobile genetic elements such as transposons or insertion sequences.

In principle, it is possible to define single nucleotide variant (SNV) thresholds to infer epidemiological links between plasmids. Aligning shared portions of homologous sequence could encompass entire plasmid sequences, specific backbone regions (a single sequence for each), or a set of non-contiguous core genes, analogous to the cgMLST approach for chromosomes [13]. However, choosing a plasmid SNV threshold involves several pragmatic considerations.

First, different plasmids from different datasets can exhibit different mutation rates. Gram-negative genera sampled from the same hospital over an 18 year period carried highly similar IncC plasmids with median 0 SNVs between backbone sequences (reference plasmid: 118kbp) [14]. In a six month period, a large European survey of *Klebsiella pneumoniae* across 32 countries found 87% of pOXA-48-like plasmids recovered were within 2 SNVs of each other (reference plasmid: 62kbp; see Fig. 1 of David *et al*.) [15]. In the same collection, pKpQIL-like (reference: 114kbp) and IncX3-like plasmids (reference: 43kbp) had congruent phylogenies with the core genome of their host strains albeit with around 10 times fewer SNVs (see Fig. 4 of David *et al*.), suggesting that the plasmid and chromosomal mutation rates were roughly similar. However, it may be necessary to account for the co-divergence of chromosomes and plasmids to accurately estimate rates of plasmid evolution, with work in *Shigella* reporting mutation rates for small plasmids up to 24 times the chromosomal rate [16].

**Fig. 1.**
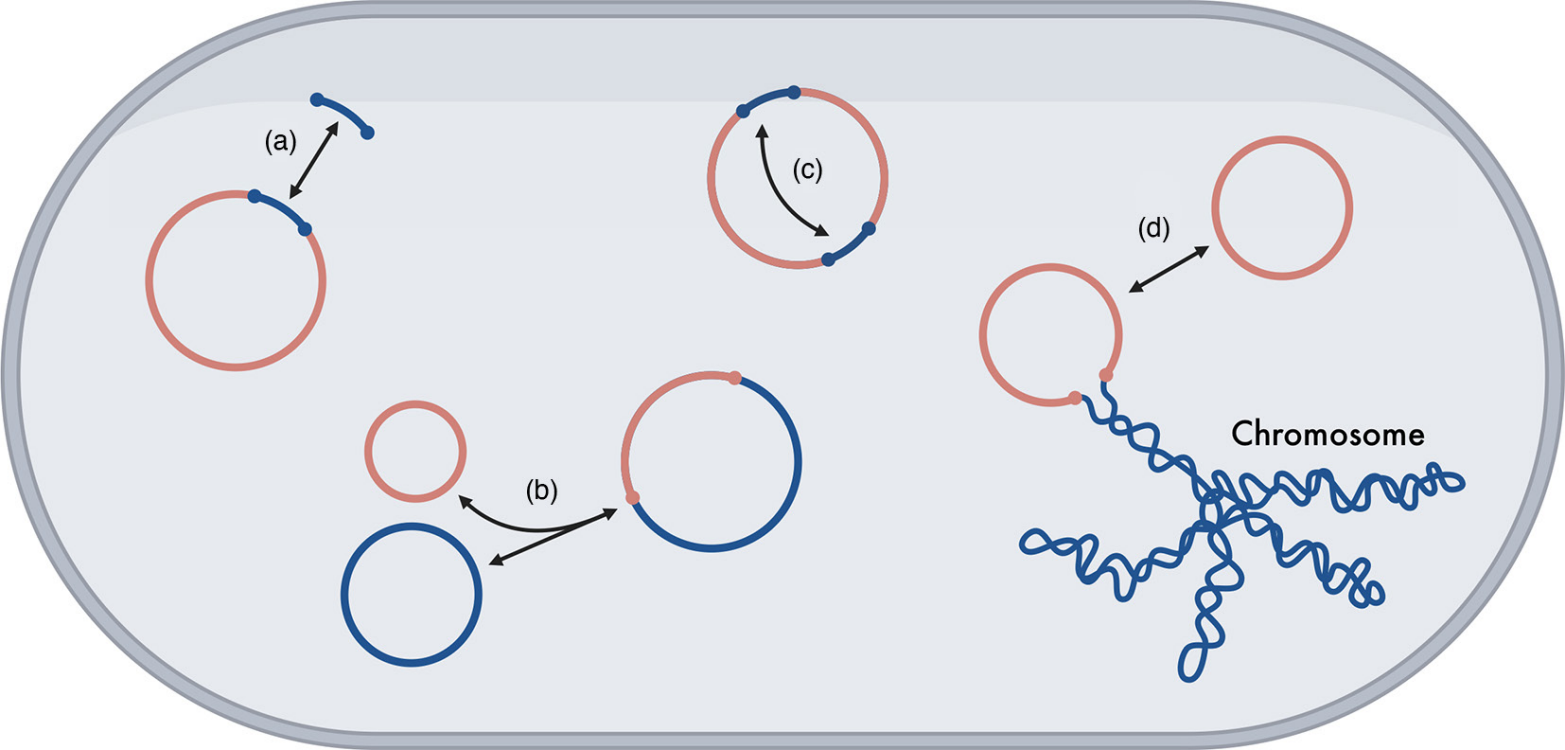
Structural changes between and within plasmids. These structural changes take place within a host cell. Arrows indicate possible changes, which might be reversible. (**a**) Gene gain and loss events within a plasmid. (**b**) Co-integration events between plasmids. (**c**) Inversions, rearrangements, recombination of sequence within a plasmid. (**d**) Chromosomal integration of plasmids. Figure made with BioRender.com.

Second, determining the appropriate regions of sequence for alignment is also difficult. Different plasmid lineages seem to exhibit different rates of gene gain and loss, thereby varying the range of genes that are shared. Col-like plasmids can remain the same length over long sampling periods (~10 years), whereas IncF-like plasmids can rapidly gain and lose genes in shorter sampling periods (~3 years) [17]. Whilst it might seem reasonable to discard genes not found in every plasmid, this might result in the loss of informative variation. In bacteria, chromosomal core genes are under a stronger purifying selection than accessory genes [18], which might indicate higher levels of polymorphism in plasmid cargo genes than their backbones. Therefore, simply counting all mutations within core genes equally to calculate an SNV distance and defining plasmids as ‘the same’ based on a distance threshold may potentially mask important cargo gene variation critical for adaptation and ecological niche specialisation. For example, in a study of clinical pOXA-48 plasmids from different patients under antibiotic treatment regimes, though 67% of plasmids were identical, there were over thirty different variants with either SNVs or insertions/deletions relative to the most common variant [19]. Experimental characterization of a subset of these plasmid variants in *E. coli* J53 showed that these variants were phenotypically significant, for example, a single missense variant in the conjugal transfer gene *traU* led to a significant drop in conjugation rate.

Third, recombination may create artificially high SNVs between plasmids that are only a few evolutionary ‘events’ apart. For plasmids with a high recombination rate, such as some IncF-like plasmids [20], removing recombination tracts is potentially important. A recent study of 162 bacterial species determined the relative rates of recombination and mutation in their chromosomes [21]. However, we currently lack such rates for plasmid backbones which would be of great help in understanding their evolution.

Fourth, plasmids vary so much in size that a single SNV threshold for small and large plasmids is unlikely to be appropriate. Size can even affect adaptation to an identical selective pressure. For example, considering the impact of restriction-modification systems, if all else is equal then smaller plasmids can more easily avoid mutational targets to evade restriction [22]. It therefore seems plausible that small plasmids are more mutationally constrained.

Finally, bacterial hosts are an important factor. A large-scale study into *E. coli* plasmids demonstrated stable lineage-associations over time of interrelated, conjugative plasmids [23]. More generally, there is the possibility of plasmid-strain-specific mutations which are not observed when the plasmid inhabits a different strain [24]. Overall, the rate and locations of mutations vary dramatically, and a sequence alignment-based approach may need to account for factors such as plasmid size, recombination rate, and host specificity. Selective effects can be largely (although not entirely) avoided by only considering four-fold degenerate sites; these are free to change to any of the other three bases without resulting in an amino-acid change, and thus are more commonly tolerant of mutations.

## Structural changes as a measure of relatedness

Alongside mutations, plasmids also diversify by undergoing ‘structural changes’ (see Fig. 1). Plasmids can gain and lose accessory genes by the action of smaller genetic elements such as transposable elements, insertion sequences, and integrons (Fig. 1a). Many plasmid lineages differ in their accessory genes, but possess the same backbone. Examples of this include IncA/C-like [25] and IncF-like [20] plasmids. Plasmids can also form co-integrates, whereby two plasmids join by homologous recombination (Fig. 1b). For example, IS*26* can form plasmid co-integrates, alongside causing deletions, inversions, and recruiting genes [26,27]. Additionally, plasmids can undergo frequent rearrangement, recombination, and inversion events, often mediated by other genetic elements [28] (Fig. 1c). Likewise, within *Klebsiella pneumoniae*, plasmids have traditionally either been virulent or resistant, but now co-integrates harbouring genes conferring both traits have been identified [29,31]. Lastly, plasmids can also be integrated and lost by the chromosome (Fig. 1d) [32]. Where multiple structural changes have impacted the genome over time, it can be very difficult or even impossible to infer individual events or the order in which they occurred.

If we still do not fully understand plasmid mutation rates, we understand far less about the rates of structural changes. The immediate impact of rearrangement events is to restrict our ability to use reference sequences. This presents a bind: core gene or *k*-mer (and other alignment-free) comparisons allow us to calculate distance measures and group plasmids. However, if we proceed with only core gene or *k*-mer comparisons, we struggle to capture any information about structural events. (Although *k*-mer comparisons may capture rearrangements, it isn’t possible to distinguish between rearrangement and nucleotide variation.) This limits our ability to trace the structural evolutionary history of plasmids, to understand the selective pressures driving structural changes, and to establish causative relationships between specific structural changes and phenotype. A recent tool pling can calculate rearrangement distances between plasmids based on a model of the number of structural events needed to turn one plasmid into another, marking an important step forward here [33].

## Future directions

Despite theoretical challenges, proceeding empirically is possible. A recent study of over 3 000 clinically-associated plasmids combined both SNVs and reference sequence coverage, thereby uniting both mutational and structural differences with a pragmatic similarity threshold: >95% of genetic content and <15 SNVs/100kbp [34]. However, it remains to be seen how well this threshold generalises, especially to plasmids which recombine regularly and lack a usable reference sequence. The new possibility of computing rearrangement distances with pling suggests that a threshold for structural events could also be determined using the same approach and dataset.

So what’s next? Future research directions include:

## Determining the relative importance and rate of mutations in plasmids

The mutation rate in plasmids can vary by selective pressure, plasmid type, and location within the plasmid. For epidemiology, a robust understanding of plasmid mutation rates – including what constitutes a plasmid ‘generation’ – and therefore appropriate phylogenetic models, is required. Moreover, it’s worth noting that effective population size (which is proportional to the rate of evolution) should be considered, as well as checking the calculated mutation rate against the expected rate of sequencing errors.

## Taking plasmid population dynamics into account

Plasmid population dynamics can be complex and very different to chromosomes. These dynamics include genetic drift through random segregation, plasmid interference which prolongs the fixation times of beneficial mutations, and genetic dominance effects which mean the rate at which new mutations are established is lower when the mutation is recessive [35]. Plasmids also often exist at multiple copies per cell relative to the chromosome. Experimentally, this means genes can evolve faster on plasmids due to the combined effect of a higher mutational target and a higher gene dosage effect for new mutations [36]. Therefore, applying similarity measures for plasmids derived from haploid organisms to polyploid plasmids requires careful reconsideration to accurately reflect their unique evolutionary dynamics.

## Determining the relative importance and rate of structural changes in plasmids

Many molecular mechanisms driving structural changes have been characterised in the literature, particularly with regard to sub-plasmid mobile genetic elements (MGEs) associated with AMR genes. As argued by Partridge *et al.* (2021), future work must build upon these decades of research [37]. The challenge is scaling bespoke workflows for small datasets to the age of big genomic data. One promising avenue employs the lack of target specificity of sub-plasmid MGEs, which can leave unique junctions in plasmid sequences. These have value as epidemiological markers, and have been used to temporally order structural changes [38,40]. Similar to mutations, structural changes might also vary in character and rate between plasmid types due to differing mechanisms and selective pressures. This includes models which quantify the relative rate of structural changes in plasmids versus mutations in the plasmid backbones. Studying these changes in natural populations will reveal the frequency and order in which they have occurred, and help establish when it is or is not appropriate to use plasmid reference sequences.

## Integrating methods for characterising plasmid structural changes into routine bacterial surveillance

Challenges remain in efficiently resolving the structural changes in plasmids for large, routine surveillance projects, which calls for the automation of several analytical steps. Centrally, we need new reference databases to catalogue core and accessory variation in plasmids. As our catalogue of plasmid diversity grows, we must acknowledge that new diversification mechanisms will be discovered with the potential to further complicate plasmid epidemiology, particularly for less-well studied microbes. For example, GR13 plasmids within *Acinetobacter* have recently been shown to have a XerC/D-mediated diversification mechanism that potentiates the accumulation and transfer of accessory genes [41,42]. This underscores the challenges that the fascinating evolutionary biology of plasmids can pose for those trying to understand their spread.

In summary, there are many open questions in quantifying plasmid similarity, many of which are entwined with the challenges we face in understanding plasmid evolution. However, recent approaches offer great promise for analysing the relationship between mutations and structural changes. It seems hopeful that, at least for well-characterised species, the near future will see the synthesis of a generalised framework that places plasmid epidemiology on a more certain footing.

## Abbreviations

AMR: antimicrobial resistance
cgMLST: core gene multilocus sequence typing
MGE: mobile genetic element
PTU: plasmid taxonomic unit
SNV: single nucleotide variant
VF: virulence factor

## References

[1] Coluzzi C, Garcillán-Barcia MP, de la Cruz F, Rocha EPC (2022). Evolution of plasmid mobility: origin and fate of conjugative and nonconjugative plasmids. Mol Biol Evol.

[2] Castañeda-Barba S, Top EM, Stalder T (2024). Plasmids, a molecular cornerstone of antimicrobial resistance in the One Health era. Nat Rev Microbiol.

[3] Carattoli A, Zankari E, García-Fernández A, Voldby Larsen M, Lund O (2014). In silico detection and typing of plasmids using plasmidfinder and plasmid multilocus sequence typing. Antimicrob Agents Chemother.

[4] Robertson J, Nash JHE (2018). MOB-suite: software tools for clustering, reconstruction and typing of plasmids from draft assemblies. Microb Genom.

[5] Redondo-Salvo S, Bartomeus-Peñalver R, Vielva L, Tagg KA, Webb HE (2021). COPLA, a taxonomic classifier of plasmids. BMC Bioinform.

[6] Acman M, van Dorp L, Santini JM, Balloux F (2020). Large-scale network analysis captures biological features of bacterial plasmids. Nat Commun.

[7] Orlek A, Phan H, Sheppard AE, Doumith M, Ellington M (2017). Ordering the MOB: Insights into replicon and MOB typing schemes from analysis of a curated dataset of publicly available plasmids. Plasmid.

[8] Schürch AC, Arredondo-Alonso S, Willems RJL, Goering RV (2018). Whole genome sequencing options for bacterial strain typing and epidemiologic analysis based on single nucleotide polymorphism versus gene-by-gene-based approaches. Clin Microbiol Infect.

[9] Mageiros L, Méric G, Bayliss SC, Pensar J, Pascoe B (2021). Genome evolution and the emergence of pathogenicity in avian *Escherichia coli*. Nat Commun.

[10] Lees JA, Harris SR, Tonkin-Hill G, Gladstone RA, Lo SW (2019). Fast and flexible bacterial genomic epidemiology with PopPUNK. Genome Res.

[11] Petrovski S, Stanisich VA (2011). Embedded elements in the IncPbeta plasmids R772 and R906 can be mobilized and can serve as a source of diverse and novel elements. Microbiology.

[12] Redondo-Salvo S, Fernández-López R, Ruiz R, Vielva L, de Toro M (2020). Pathways for horizontal gene transfer in bacteria revealed by a global map of their plasmids. Nat Commun.

[13] Cody AJ, Bray JE, Jolley KA, McCarthy ND, Maiden MCJ (2017). Core genome multilocus sequence typing scheme for stable, comparative analyses of *Campylobacter jejuni* and *C. coli* human disease isolates. J Clin Microbiol.

[14] Macesic N, Hawkey J, Vezina B, Wisniewski JA, Cottingham H (2023). Genomic dissection of endemic carbapenem resistance reveals metallo-beta-lactamase dissemination through clonal, plasmid and integron transfer. Nat Commun.

[15] David S, Cohen V, Reuter S, Sheppard AE, Giani T (2020). Integrated chromosomal and plasmid sequence analyses reveal diverse modes of carbapenemase gene spread among *Klebsiella pneumoniae*. Proc Natl Acad Sci U S A.

[16] Müller NF, Duchêne S, Williamson DA, Howden BP, Ingle DJ (2022). Tracking the horizontal transfer of plasmids in *Shigella sonnei* and *Shigella flexneri* using phylogenetics. bioRxiv.

[17] Matlock W, Lipworth S, Chau KK, AbuOun M, Barker L (2023). Enterobacterales plasmid sharing amongst human bloodstream infections, livestock, wastewater, and waterway niches in Oxfordshire, UK. elife.

[18] Steinberg AP, Lin M, Kussell E (2022). Core genes can have higher recombination rates than accessory genes within global microbial populations. eLife.

[19] DelaFuente J, Toribio-Celestino L, Santos-Lopez A, León-Sampedro R, Alonso-Del Valle A (2022). Within-patient evolution of plasmid-mediated antimicrobial resistance. Nat Ecol Evol.

[20] Matlock W, Chau KK, AbuOun M, Stubberfield E, Barker L (2021). Genomic network analysis of environmental and livestock F-type plasmid populations. ISME J.

[21] Torrance EL, Burton C, Diop A, Bobay L-M (2024). Evolution of homologous recombination rates across bacteria. Proceed Nat Acad Sci.

[22] Shaw LP, Rocha EPC, MacLean RC (2023). Restriction-modification systems have shaped the evolution and distribution of plasmids across bacteria. Nucleic Acids Res.

[23] Arredondo-Alonso S, Pöntinen AK, Gama JA, Gladstone RA, Harms K (2023). *Escherichia coli* plasmidome maps the game of clones. bioRxiv.

[24] Benz F, Hall AR (2023). Host-specific plasmid evolution explains the variable spread of clinical antibiotic-resistance plasmids. Proc Natl Acad Sci U S A.

[25] Hegyi A, Szabó M, Olasz F, Kiss J (2017). Identification of oriT and a recombination hot spot in the IncA/C plasmid backbone. Sci Rep.

[26] Varani A, He S, Siguier P, Ross K, Chandler M (2021). The IS6 family, a clinically important group of insertion sequences including IS26. Mob DNA.

[27] Harmer CJ, Hall RM (2024). IS 26 and the IS 26 family: versatile resistance gene movers and genome reorganizers. Microbiol Mol Biol Rev.

[28] Dionisio F, Zilhão R, Gama JA (2019). Interactions between plasmids and other mobile genetic elements affect their transmission and persistence. Plasmid.

[29] Lam MMC, Wyres KL, Wick RR, Judd LM, Fostervold A (2019). Convergence of virulence and MDR in a single plasmid vector in MDR *Klebsiella pneumoniae* ST15. J Antimicrob Chemother.

[30] Peter S, Bosio M, Gross C, Bezdan D, Gutierrez J (2020). Tracking of antibiotic resistance transfer and rapid plasmid evolution in a hospital setting by nanopore sequencing. mSphere.

[31] Shelenkov A, Mikhaylova Y, Voskanyan S, Egorova A, Akimkin V (2023). Whole-genome sequencing revealed the fusion plasmids capable of transmission and acquisition of both antimicrobial resistance and hypervirulence determinants in multidrug-resistant *Klebsiella pneumoniae* isolates. Microorganisms.

[32] Smith TJ, Tian R, Imanian B, Williamson CHD, Johnson SL (2021). Integration of complete plasmids containing bont genes into chromosomes of *Clostridium parabotulinum*, *Clostridium sporogenes*, and *Clostridium argentinense*. Toxins.

[33] Frolova D, Lima L, Roberts L, Bohnenkämper L, Wittler R (2024). Applying rearrangement distances to enable plasmid epidemiology with pling. Bioinformatics.

[34] Evans D, Sundermann A, Griffith M, Rangachar Srinivasa V, Mustapha M (2023). Empirically derived sequence similarity thresholds to study the genomic epidemiology of plasmids shared among healthcare-associated bacterial pathogens. EBioMedicine.

[35] Rodríguez-Beltrán J, DelaFuente J, León-Sampedro R, MacLean RC, San Millán Á (2021). Beyond horizontal gene transfer: the role of plasmids in bacterial evolution. Nat Rev Microbiol.

[36] San Millan A, Escudero JA, Gifford DR, Mazel D, MacLean RC (2016). Multicopy plasmids potentiate the evolution of antibiotic resistance in bacteria. Nat Ecol Evol.

[37] Partridge SR, Enne VI, Grohmann E, Hall RM, Rood JI (2021). Classifying mobile genetic elements and their interactions from sequence data: the importance of existing biological knowledge. Proc Natl Acad Sci U S A.

[38] Ambrose SJ, Harmer CJ, Hall RM (2018). Evolution and typing of IncC plasmids contributing to antibiotic resistance in Gram-negative bacteria. Plasmid.

[39] Hu Y, Moran RA, Blackwell GA, McNally A, Zong Z (2022). Fine-scale reconstruction of the evolution of FII-33 multidrug resistance plasmids enables high-resolution genomic surveillance. mSystems.

[40] Moran RA, Behruznia M, Holden E, Garvey MI, McNally A (2024). PQEB1: a hospital outbreak plasmid lineage carrying blakpc-2. bioRxiv.

[41] Moran RA, Liu H, Doughty EL, Hua X, Cummins EA (2022). GR13-type plasmids in *Acinetobacter* potentiate the accumulation and horizontal transfer of diverse accessory genes. Microb Genom.

[42] Ambrose SJ, Hall RM (2024). Variation in the plasmid backbone and dif module content of R3-T33 Acinetobacter plasmids. Plasmid.

