# Erratum: Towards quantifying plasmid similarity

**DOI:** 10.1099/mgen.0.001329

**Published:** 2024-12-13

**Authors:** William Matlock, Liam P. Shaw, Samuel K. Sheppard, Edward Feil

**Affiliations:** 1 Nuffield Department of Medicine, University of Oxford, Oxford, UK; 2 Department of Biology, University of Oxford, Oxford, UK; 3 Milner Centre for Evolution, Department of Life Sciences, University of Bath, Bath, UK

In the published version of this article, there was a mistake in the reference list.

Reference number 33 listed the incorrect journal name and previously appeared as:

33. Frolova D, Lima L, Roberts L, Bohnenkämper L, Wittler R *et al.* Applying rearrangement distances to enable plasmid epidemiology with pling. *Bioinformatics* 2024

The correct reference should have appeared as:

33. Frolova, D *et al.* Applying rearrangement distances to enable plasmid epidemiology with pling. *bioRxiv* 2024.06.12.598623 (2024) doi:10.1101/2024.06.12.598623

The reference in the original published version has been corrected.

The Microbiology Society apologizes for any inconvenience caused.

